# Chemical Composition and Biological Activity of the Essential Oil from Leaves of *Moringa oleifera* Lam. Cultivated in Mozambique

**DOI:** 10.3390/molecules180910989

**Published:** 2013-09-09

**Authors:** Tatiana Marrufo, Filomena Nazzaro, Emilia Mancini, Florinda Fratianni, Raffaele Coppola, Laura De Martino, Adelaide Bela Agostinho, Vincenzo De Feo

**Affiliations:** 1 Centre for Research and Development in Ethnobotany–CIDE, Rua de Igreja, Casa zero, Vila Namaacha, Maputo Province, Mozambique; E-Mails: tatiana_marrufo@hotmail.com (T.M.); bela_3105@hotmail.com (A.B.A.); 2 Institute of Food Science, CNR-ISA, Via Roma 64, 83100 Avellino, Italy; E-Mails: mena@isa.cnr.it (F.N.); fratianni@isa.cnr.it (F.F.); direttore@isa.cnr.it (R.C.); 3 Department of Pharmacy, University of Salerno, Via Giovanni Paolo II, 132, 84084 Fisciano (Salerno), Italy; E-Mails: emancini@unisa.it (E.M.); ldemartino@unisa.it (L.D.M.)

**Keywords:** *Moringa oleifera*, essential oil, antimicrobial activity, antioxidant activity

## Abstract

The antioxidant capacity and antimicrobial activity of the essential oil of *Moringa oleifera* (*Moringaceae*) grown in Mozambique was investigated. The chemical composition was studied by means of GC and GC-MS analysis. Hexacosane (13.9%), pentacosane (13.3%) and heptacosane (11.4%) were the main components. Ultra High Performance Chromatography-DAD analysis detected the flavonoids quercetin (126 μg/g) and luteolin (6.2 μg/g). The essential oil exhibited a relatively low free radical scavenging capacity. The antimicrobial activity of the essential oil was assayed against two Gram-positive strains (*Bacillus cereus*, *Staphylococcus aureus*), two Gram-negative strains (*Escherichia coli*, *Pseudomonas aeruginosa*), and five fungal strains of agro-food interest (*Penicillium aurantiogriseum*, *Penicillium expansum*, *Penicillium citrinum*, *Penicillium* *digitatum*, and *Aspergillus niger* spp.). *B. cereus* and *P. aeruginosa*, as well as the fungal strains were sensitive to the essential oil.

## 1. Introduction

*Moringa*, native to Asia and spread in most parts of Africa, is the sole genus in the flowering plant family *Moringaceae*. This genus is made of 12 species [1]. *Moringa oleifera* Lam. is one of the most economically important species indigenous to dry tropical areas in the Northwestern India, at the Southwestern foot of the Himalayas [2]. Moreover, it is widely cultivated in different countries [3]. An extensive variety of nutritional and medicinal uses have been attributed to its roots, bark, leaves, flowers, fruits and seeds [4]. Almost all parts of this plant have been used for various diseases in the folk medicine of South Asia, including the treatment of inflammation and infectious diseases along with cardiovascular, gastrointestinal, haematological and hepatic and kidney disorders [3,4]. Leaves of *M. oleifera* are traditionally used as purgatives and in the treatment of headaches, haemorrhoids, fevers, inflammation of noise and throat, bronchitis, eye and ear infections, and to combat vitamin C deficiency. The leaf juice is believed to control glycaemia and is applied for swollen glands. Leaves of *M. oleifera* are cooked and eaten like spinach or used to prepare soups and salads. Fresh leaves have been reported to contain vitamin C and vitamin A, more than those reported in carrots and oranges [5]. They are also used to contrast hypertension and cholesterol; indeed, anticancer, antitumor, anti-inflammatory, diuretic properties as well as antihepatotoxic, antifertility, antiurolithiatic and analgesic activities were reported [6]. *M. oleifera* is also known for its antioxidant activity, essentially due to the presence of high amounts of polyphenols [3].

However, very little is known in literature about the composition of the leaf essential oil and its antimicrobial properties [5,7,8]. The antifungal activity of crude extracts and essential oil of *M. oleifera* against *Trichophyton rubrum*, *T. mentagrophytes*, *Epidermophyton xoccosum*, and *Microsporum canis* has been recently reported [7]. Therefore, the aim of this paper was to study the chemical composition of the essential oil obtained from leaves of *M. oleifera* grown in Mozambique and to evaluate its potential antioxidant and *in vitro* antimicrobial activity*.*

## 2. Results and Discussion

### 2.1. Chemical Composition of the Essential Oils

The hydrodistillation of the leaves of *M. oleifera* produced a pale yellow oil in 0.05% yield on a dry mass basis. Table 1 shows the chemical composition of the essential oil; compounds are listed according to their elution order on a HP-5MS column.

In all, 29 compounds were identified, accounting for 92.3% of the total oil, and hydrocarbons represented the 91.1% of the oil. Hexacosane (13.9%), pentacosane (13.3%) and heptacosane (11.4%) were the most abundant compounds. Such a composition is similar to that of the oil obtained from leaves of *M. oleifera* grown in Taiwan, in which pentacosane (17.4%), hexacosane (11.2%) and (*E*)-phytol (7.7%) were the major components [7]. Phytol (21.6%) and thymol (9.6%) have been reported as the most abundant compounds in the leaves oil of *M. oleifera* from Ceará (Brazil) [8].

Mukunzi and coworkers [5] reported the comparison of volatile profile of *M. oleifera* leaves from Rwanda and China. The Rwandan sample contained 59 compounds, with hexanoic acid (19.8% of total volatiles) as the most abundant one, while the Chinese sample contained mainly acetic acid (12.5% of total volatiles).

Nonacosane (18.6%), 1,2,4-trimethyl-benzene (16.9%) and heptacosane (7.4%) were the major components in the essential oil of *M. oleifera* obtained by Soxhlet extraction, while nonacosane (13.4%–60.1%), heptacosane (5.0%–22.6%) and pentacosane (1.0%–6.3%) were among the most abundant components in the essential oil obtained by Supercritical Fluid Extraction under different conditions [9].

**Table 1:** Chemical composition of the leaf essential oil from *Moringa oleifera.*

Component	Ri ^a^	Ri ^b^	Identification ^c^	%
**Oxygenated monoterpenes**				
Linalool	1099	1553	1,2,3	t
α-Terpineol	1189	1706	1,2,3	t
**Phenolic compounds**				
*p*-Vinylguaiacol	1311	1937	1,2	t
**Oxygenated sesquiterpenes**				0.7
*cis*-Dihydroagarofuran	1518		1,2	0.1
Eudesm-11-en-4-α,6α-diol	1807		1,2	0.6
**Hydrocarbons**				91.1
1-Octadecene	1783		1,2	0.3
Octadecane	1800		1,2,3	0.1
5-Octadecin	1844		1,2	0.3
*n*-Hexadecanol	1889		1,2	0.1
Nonadecane	1896		1,2,3	0.8
1-Eicosene	1990		1,2	0.3
Eicosane	1998		1,2,3	1.2
*n*-Octadecanol	2091		1,2	0.2
Heneicosane	2100		1,2,3	1.9
Cyclopentadecanol	2119		1,2	0.4
1-Docosene	2191		1,2	0.4
Docosane	2200		1,2,3	6.8
*cis*-9-Eicosen-1-ol	2224		1,2	0.3
Tricosane	2297		1,2,3	8.1
Tetracosane	2405	2400	1,2,3	9.7
Pentacosane	2499	2500	1,2,3	13.3
Hexacosane	2601	2600	1,2,3	13.9
Heptacosane	2698	2700	1,2,3	11.4
Octacosane	2821	2800	1,2,3	10.0
Nonacosane	2930	2900	1,2,3	10.5
Triacontane	3008	3000	1,2,3	1.1
**Others**				0.5
Hexenyl propanoate	1101		1,2	t
Phenylethyl alcohol	1110		1,2	t
Pseudo Phytol	2016		1,2	0.5
Total identified				92.3

^a^ Kovats retention index on HP-5 MS column; ^b^ Kovats retention index on HP Innowax;

^c^ 1 = Kovats retention index, 2 = mass spectrum, 3 = co-injection with authentic compound; t = trace, less than 0.1%.

### 2.2. UPLC-DAD Analyses

**Figure 1:**
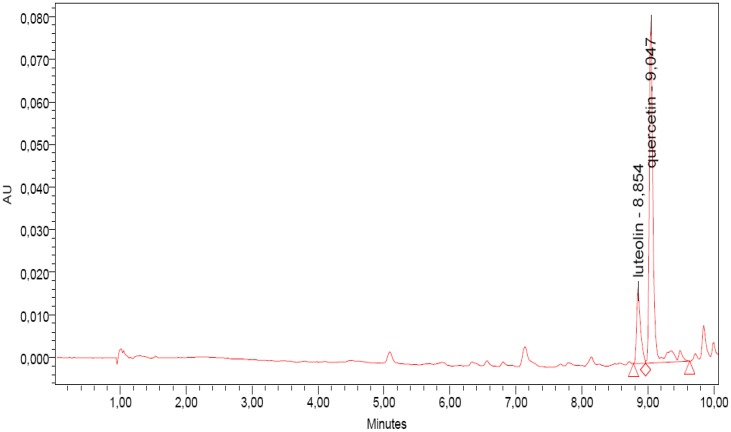
UPLC chromatogram of the essential oil of *M. oleifera.* For details, see Experimental.

UPLC-DAD analysis revealed two main peaks, namely quercetin and luteolin, identified by the elution times of the respective standards (Figure 1). Quercetin is generally detected as the main component of the hydro-alcoholic extract obtained from *M. oleifera*. No kaempferol, nor crypto-chlorogenic acid, isoquercetin and astragalin, which are main antioxidant components in the leaves of this plant were found [10,11]. Therefore, the analytical approach was carried out on the essential oil, a structural matrix well known more obviously abundant of other components than of polyphenols. Generally, the presence of polyphenols in the essential oils is not frequent but it is not exceptional either. Some authors have reported the liquid chromatographic analysis performed on essential oils, which detected different compounds having antioxidant capacity.

Ly and coworkers [12] ascertained, through HPLC-DAD-MS performed using a C_18_ column, that the antioxidant activity of the essential oils of *Angelica sinensis* Oliv. (Diels) could be ascribable to the presence of coniferyl ferulate. Luteolin and quercetin were identified, with many other metabolites, in virgin olive oil, a model of a lipidic matrix, after pre-extraction of the polyphenols mixture with methanol and subsequent analysis by HPLC-UV with a C_18_ column [13]. In this research, a chromatographic analysis of the polyphenol profile of the *M. oleifera* essential oil was performed by UPLC-DAD for the first time.

### 2.3. Antioxidant Activity

The EC_50_ value of the essential oil, that is the amount of the essential oil necessary to inhibit the activity of DPPH of 50%, was 30.28 mg. *M. oleifera* leaves act as a good source of natural antioxidants due to the presence of various types of antioxidant compounds such as ascorbic acid, flavonoids, phenolics and carotenoids [14,15]. The presence of the two flavonoids with proven antioxidant capability might give a further added value to the essential oil, due to their potentially anticarcinogenic properties, a peculiarity reported for several fruits and vegetables [16,17]. The detection of quercetin in the essential oil of *M. oleifera* is of particular interest.

Quercetin contains phenolic hydroxy groups with antioxidant action with potential therapeutic uses [18]. In fact, *in vitro* studies have ascertained that quercetin like other flavonoids may strongly inhibit the production of both nitric oxide and tumor necrosis factor by Kupffer cells when stimulated by injury [19]. Quercetin also protects cells against injury caused by X-rays, and may act in preventing carcinogenesis [20]. Luteolin has also a strong antioxidant activity and may exhibit a protective capability on DNA [21]. Preclinical studies have shown that luteolin is capable of exerting a multiplicity of biological and pharmacological activities, including antioxidant, anti-inflammatory, antimicrobial, anticancer, anti-allergic, anti-platelet, and other activities [22]. 

The hydrocarbons found in the essential oil of *M. oleifera* could have also antioxidant activity, as demonstrated in other essential oils. The essential oil of *Rosa damascena*, rich in hexatriacontane (24.6%), 1-nonadecene (18.56%), and *n*-tricosane (16.68%), showed stronger radical scavenging effect [23]. A seed hexane extract of *Hypericum scabrum* L., containing omega-3 fatty acids (37.6%), a bis (2-ethylhexyl)phthalate (35.7%), linoleic acid (6.9%) and nonacosane (3.9%) as major compounds, showed high radical scavenging activity [24].

### 2.4. Antibacterial and Antifungal Activity

Using the inhibition halo technique, the antimicrobial activity of the essential oil of *M. oleifera* was evaluated using different amount of total polyphenols against *Bacillus cereus* and *Staphylococcus aureus* (Gram-positive strains), and *Escherichia coli* and *Pseudomonas aeruginosa* (Gram-negative strains). The antifungal activity was evaluated assaying the swame amount of polyphenols against *Penicillium aurantiogriseum*, *Penicillium expansum*, *Penicillium citrinum*, *Penicillium* *digitatum* and *Aspergillus niger*. The results are reported in Table 2.

Generally, the degree of the essential oil activity is revealed by the size of inhibition zone that is expressed by the diameter of the referred inhibition zone. Due to the simple nature of this assay and the reduced amount of essential oil required, the use of this technique is generally recommended also for the evaluation of the essential oils. This technique, although less suitable for more precise quantification purposes, such as the determination of the MIC values, is also used to determine the susceptibility of a range of microbial species to a particular essential oil [25].

*B. cereus* was the most sensitive strain: an inhibition halo of 5.7 mm was observed just by using 2 µg/plate of essential oil. *P. aeruginosa* was inhibited by 5 µg/plate of essential oil.

It is well known that essential oils and their components show a better antimicrobial effectiveness against Gram-positive bacteria. Due to the composition of outer membrane, essential oil can alter not only such structures but penetrate within the cell, leading to those alterations, such as the denaturation of proteins and enzymes, the “unbalance” of the K^+^ and H^+^ ion concentration, until the modification of the entire cell morphology, which can lead to the death of the microorganism. Phenolic compounds show generally a good antimicrobial effectiveness against Gram-positive bacteria; their effect is dependent on their amount: at low concentrations they are able to interfere with enzymes involved in the production of energy; at higher concentrations, they can induce the denaturation of proteins [26] until an irreversible modification of the cell and death.

**Table 2:** Antimicrobial activity of the leaf essential oil of *Moringa oleifera*.

Organism	Doses		
	2 µg	5 µg	10 µg
**Bacteria**			
**Gram-positive strains**			
*Bacillus cereus*	5.7 ± 1.2 **	9 ± 1.7 ***	10.7 ± 1.2 ***
*Sthaphylococcus aureus*	0.0 ± 0.0	0.0 ± 0.0	0.0 ± 0.0
**Gram-negative strains**			
*Escherichia coli*	0.0 ± 0.0	0.0 ± 0.0	6.7 ± 0.6 ***
*Pseudomonas aeruginosa*	0.0 ± 0.0	5.0 ± 0.0 ***	8.0 ± 1.3 ***
**Fungal strains**			
*Penicillium aurantiogriseum*	0.0 ± 0.0	4.7 ± 0.6 ***	9.3 ± 0.6 ***
*Penicillium expansum*	5.0 ± 0.0	5.3 ± 0.3 ***	7.3 ± 0.6 ***
*Penicillium citrinum*	4.8 ± 0.3 ***	5.0 ± 0.0 ***	8.3 ± 1.2 ***
*Penicillium digitatum*	4.7 ± 0.6 ***	5.2 ± 0.3 ***	9.3 ± 0.3 ***
*Aspergillus niger*	3.7 ± 0.3 ***	5.3 ± 0.6 ***	8.8 ± 0.7 ***

Data are expressed in mm and do not include the diameter of paper disc (5 mm). Chloramphenicol (10 µg) and dimethylsulfoxide (DMSO) were used as positive and negative control, respectively. Results are shown as mean ± SD (n = 3). * *p* < 0.05, ** *p* < 0.01, *** *p* < 0.001 *vs.* DMSO.

The hydrophobicity, typical of the essential oils, represents the principal property responsible for the distribution in bacterial structures, mainly in Gram-positive bacteria. This aspect is of real and relevant importance: in fact, the permeability barrier role of cell membranes is indispensable to many cellular functions, including the maintenance of the energy status of the cell, the membrane-coupled energy-transducing processes, the solute transport and regulation of metabolism, and is also essential to control the turgor pressure [27].

On the other hand, the Gram-negative *E. coli*, although more resistant, was inhibited with an inhibition halo of 6.7 mm. The molecular mechanism of action of the essential oil of *M. oleifera* is unknown, but the essential oil can probably inhibit the generation of adenosine triphosphate from dextrose and disrupt the cell membrane [28]. The high amount of hydrocarbons and the concurrent presence of quercetin and luteolin could also contribute to inhibit the microbial DNA gyrase [29]. Conversely, the Gram-positive *S. aureus* was the most resistant strain. This disagrees with the literature [30] that reported a decline of the intracellular ATP content in *S. aureus* and a subsequent decline of the bacterial growth in the presence of the oregano essential oil. Probably, the composition and the synergy among the different biomolecules present in *M. oleifera* essential oil acted in different manner.

The extracts from *M. oleifera* could be an effective source of natural antimicrobials with versatile potential applications, for instance in the pharmaceutical industry to control different Gram-positive and Gram-negative bacteria, also involved in gastrointestinal diseases [31,32,33]. Despite the many traditional antimicrobial uses of *M. oleifera*, scant literature is available on its possible uses as sanitizer or preservative in foods. Therefore, a very important step in the screening of a plant material for preservative activity is to evaluate its antimicrobial activity against different microorganisms [34,35] with the aim to hypothesize its potential use as natural preservative agent, to be used in the health and food industry.

Essential oil of *M. oleifera* showed different degrees of antifungal activity. The essential oil showed inhibitory activity against all fungi used as test strains. The antifungal activity resulted of particular meaning, taking mainly into account the very low doses used in the test, ranging from 2 µg/disk to 10 µg/disk, and the fungal tester strains, all of agro-food and health interest. All strains were sensitive to the essential oil at 2 µg/disk, but *Aspergillus niger* was sensitive to the essential oil at the higher concentrations, giving a similar inhibition halo. A citrinin-producing strain (*Penicillium citrinum*) was inhibited using only at 2 µg/disk, showing an inhibition halo of 4.8 mm. The high amount of hydrocarbons found in our sample may be responsible for the antifungal activity, in agreement with the literature [35].

## 3. Experimental Section

### 3.1. Plant Material

The leaves of *M. oleifera* were collected in September 2012 at Xai-Xai, Gaza Province, Mozambique, on the farm of the Regional Center of Science and Technology. Leaves were dried in shade and pulverized with a slicer type baby serial 5244 at the Center for Research and Development in Ethnobotany. The plant was identified by Dr. Filomena Barbosa and a voucher specimen (325/2012) was deposited at the Herbarium of the Faculty of Biological Science of the University Eduardo Mondlane (Mozambique). Before extraction, the vegetal material was stored in paper bags for one week and then extracted.

### 3.2. Isolation of the Volatile Oil

One hundred grams of dried leaves of sample were grounded in a blender and then subjected to hydrodistillation for 3 h according to the standard procedure described in the *European Pharmacopoeia* (2004) [36]. The oil was solubilised in *n*-hexane, dried over anhydrous sodium sulphate and stored using stream of nitrogen at +4 °C in the dark until tested and analyzed the day after dry material gave yellow oil in a yield of 0.05% (v/w) for *M. oleifera.*

### 3.3. GC-FID Analysis

The GC-FID analysis was carried out on a Perkin-Elmer Sigma-115 gas chromatograph equipped with a flame ionization detector (FID) and a data handling processor. The separation was achieved using an apolar HP-5 MS fused-silica capillary column (30 m × 0.25 mm i.d., 0.25 μm film thickness). Column temperature: 40 °C, with 5 min initial hold, and then to 270 °C at 2 °C/min, finally at 270 °C for 20 min; injection mode splitless (1 μL of a 1:1,000 *n*-pentane solution). Injector and detector temperatures were 250 °C and 290 °C, respectively. Analysis was also run by using a fused silica HP Innowax polyethylenglycol capillary column (50 m × 0.20 mm i.d., 0.25 μm film thickness). In both cases, helium was used as carrier gas (1.0 mL/min).

### 3.4. GC/MS Analysis

The GC/MS analysis was performed on an Agilent 6850 Ser. II apparatus, fitted with a fused silica DB-5 capillary column (30 m × 0.25 mm i.d., 0.33 μm film thickness), coupled to an Agilent Mass Selective Detector MSD 5973; ionization energy voltage 70 eV; electron multiplier voltage energy 2000 V. Mass spectra were scanned in the range 40–500 amu, scan time 5 scans/s. Gas chromatographic conditions were as reported in the previous paragraph; transfer line temperature, 295 °C.

### 3.5. Identification of the Essential Oil Components

Most constituents were identified by gas chromatography by comparison of their Kovats retention indices (Ri) [determined relative to the *t*_R_ of *n-*alkanes (C_10_–C_35_)], with either those of the literature [37,38,39,40] and mass spectra on both columns with those of authentic compounds available in our laboratories by means NIST 02 and Wiley 275 libraries [41]. The components’ relative concentrations were obtained by peak area normalization. No response factors were calculated.

### 3.6. UPLC Analyses

Ultra high-performance liquid chromatography (UPLC) analysis of the polyphenols in the essential oil of *M. oleifera* was performed using an ACQUITY Ultra Performance LCTM system linked to a PDA 2996 photodiode array detector (Waters, Milford, MA, USA). The UV detection wavelength was set at 280 nm. Empower software (Waters) was used to control the instruments and for data acquisition and processing. The analysis was carried out at 30 °C using a reversed-phase column (BEH C_18_, 1.7 µm, 2.1 mm × 100 mm, Waters). The mobile phase consisted of Solvent A (7.5 mM acetic acid) and Solvent B (acetonitrile) at a flow rate of 250 μL/min. Gradient elution was employed, starting at 5% B for 0.8 min, 5%–20% B for 5.2 min, isocratic 20% B for 0.5 min, 20%–30% B for 1 min, isocratic 30% B for 0.2 min, 30%–50% B for 2.3 min, 50%–100% B for 1 min, isocratic 100% B for 1 min, and finally 100%–5% B for 0.5 min. At the end of this sequence, the column was equilibrated under the initial conditions for 2.5 min. The pressure ranged from 6000 to 8000 psi during the chromatographic run. The effluent was introduced to an LC detector (scanning range 210–400 nm, resolution 1.2 nm). The injection volume was 5 μL. Phenolic compounds were identified and quantified by comparing the peak areas on the chromatograms of samples with those of diluted standard solutions, as reported in Fratianni *et al.* [42].

### 3.7. Free Radical-Scavenging Capacity

The free radical-scavenging activity of the essential oil of *M. oleifera* was measured using the stable radical 2,2-diphenyl-1-picrylhydrazyl (DPPH) assay [43]. The analysis was performed in microplates by adding 7.5 μL of essential oil to 303 μL of a methanolic DPPH solution (153 mM). The absorbance was then spectrophotometrically measured (Varian Cary 50 MPR, Cernusco sul Naviglio (MI), Italy) at λ = 517 nm. The absorbance of DPPH without antioxidant (control sample) was used as the baseline measurement.

The scavenging activity was expressed as the 50% effective concentration (EC_50_), which was defined as the sample concentration (μg/mL) necessary to inhibit DPPH radical activity by 50% after a 60 min incubation. These experiments were performed in triplicate, and the results are expressed as the mean ± standard deviation.

### 3.8. Antibacterial and Antifungal Activity

The inhibition halos test on agar plates was employed to investigate the antibacterial activity of the essential oil of *M. oleifera* [44]. Different concentrations of the essential oil of *M. oleifera* were assayed against the following pathogens: Gram-positive strains *Bacillus cereus* DSM 4313, *Staphylococcus aureus* DSM 25923; Gram-negative strains *Escherichia coli* DSM 8579 and *Pseudomonas aeruginosa* ATCC 50071 All strains were purchased from Deutsche Sammlung von Mikroorganismen und Zellkulturen GmbH (DSMZ, Braunschweig, Germany). Each strain was incubated at 37 °C for 18 h in Nutrient Broth (Sigma, Milan, Italy). The microbial suspensions (1 × 10^8^ Colony Forming Units-CFU-/mL) were uniformly spread onto the solid media plates (Ø = 90 mm dishes). The surface of the inoculated plate was spotted with different amounts of the essential oil, previously diluted 1:10 (*v/v*) in dimethylsulfoxide (DMSO). After 30 min under sterile conditions at room temperature, plates were incubated at 37 °C for 24–48 h, depending on the strain. The diameter of the clear zone shown on plates was accurately measured and the antibacterial activity expressed in mm. Sterile ultrapure water and DMSO (10 μL/paper disc) were used as negative control. Chloramphenicol suspended in physiological solution (66 μg/spot solution), served as positive controls. Samples were tested in triplicate and results are expressed as mean ± standard deviation.

The potential antifungal activity was evaluated using as tester microorganisms five fungal strains of agro-food interest, *Penicillium aurantiogriseum* DSM 2429, *Penicillium expansum* DSM 1994, *Penicillium citrinum* DSMZ 1179, *Penicillium digitatum* DSMZ 2749, and *Aspergillus niger* spp., were used. All strains were purchased from DSMZ.

The strains were grown in potato dextrose broth (Sigma, Milan, Italy at 28 °C). A cell suspension of fungi was prepared in sterile distilled water, adjusted to contain approximately 10^6^ CFU/mL, and plated onto Potato Dextrose Agar (PDA). Different amounts of the essential oil previously diluted 1:10 (*v/v*) in DMSO were used. After 30 min under sterile conditions, the inoculated plates were spotted with different amount of the essential oil, diluted as above described. After 20 min under sterile conditions at room temperature, plates were incubated at 28 °C for 48–72 h. When the mycelium of fungi reached the edges of the control plate (negative control without the samples added), the diameter of the clear zone shown on plates was accurately measured (“Extra steel caliper mod 0289”, mm/inch reading scale, precision 0.05 mm, Mario De Maio, Milan, Italy) and the antifungal activity expressed in mm. DMSO was used as negative control (10 μL/paper disc). Samples were tested in triplicate and the results are expressed as mean ± standard deviation.

## 4. Conclusions

Data obtained in this work could be useful in determining the chemical characteristics of the leaf essential oil of *M. oleifera*, currently little known. Data on antioxidant and antimicrobial activities can contribute to confirm the popular uses of this plant in Mozambique folk practices and to suggest new practical uses of the plant derivatives.
